# A defined human aging phenome

**DOI:** 10.18632/aging.102166

**Published:** 2019-08-12

**Authors:** Søren Norge Andreassen, Michael Ben Ezra, Morten Scheibye-Knudsen

**Affiliations:** 1 Center for Healthy Aging, Department of Cellular and Molecular Medicine University of Copenhagen, Copenhagen, Denmark

**Keywords:** aging, phenotype, phenome, data mining

## Abstract

Aging is among the most complex phenotypes that occur in humans. Identifying the interplay between different age-associated features is undoubtedly critical to our understanding of aging and thus age-associated diseases. Nevertheless, what constitutes human aging is not well characterized. Towards this end, we mined millions of PubMed abstracts for age-associated terms, enabling us to generate a detailed description of the human aging phenotype. We discovered age-associated features in clusters that can be broadly associated with previously defined hallmarks of aging, consequently identifying areas where interventions could be pursued. Importantly, we validated the newly discovered features by manually verifying the prevalence of these features in combined cohorts describing 76 million individuals, allowing us to stratify features in aging that appear to be the most prominent. In conclusion, we propose a comprehensive landscape of human aging: the human aging phenome.

## Introduction

Aging represents the largest risk factor for chronic diseases and a significant and growing socioeconomic challenge for most societies worldwide. Nevertheless, what constitutes the human phenotype of aging is not well characterized, likely due to the highly complex and heterogeneous nature of human aging. Indeed, aging is probably caused by the stochastic failure of a myriad of different biological processes leading to increased susceptibility to disease and death [1].

Due to the role of aging in numerous diseases, interventions leading to healthy aging are being heavily investigated. Clinical trials for aging interventions are challenging due to the possibility of long trial times and/or the necessity to investigate large cohorts. The generation of biomarkers that may predict the age and health of an individual has therefore received significant interest. Importantly, several recent breakthroughs have allowed us to discover complex biomarkers, or aging clocks, which are able to predict the age and risk of death and/or age-associated disease of individuals [2–6]. Nevertheless, it is unclear how these biomarkers predict the multitude of phenotypes associated with aging. To this end, having a well-defined phenotypical description of human aging and an understanding of how different aging phenotypes associate with each other will enable us to better understand aging, design trials and discover drugs targeting the aging process.

Herein, we used a previously incomplete list of phenotypes associated with human aging to mine millions of PubMed articles for co-occurring phenotypes, allowing us to better define what we term the human aging phenome. We used this computationally unbiased approach to generate a list of approximately a thousand terms and then manually curated this list to extract features associated with aging. We then validated these features manually against the description of more than 75 million individuals from published studies. Notably, these parameters cover all tissues in the human body and illustrate the heterogeneity of the human aging phenotype. Collectively, our results allow us to propose a description of what human aging is.

## RESULTS

### Identification of abstracts describing human aging

**Figure 1:**
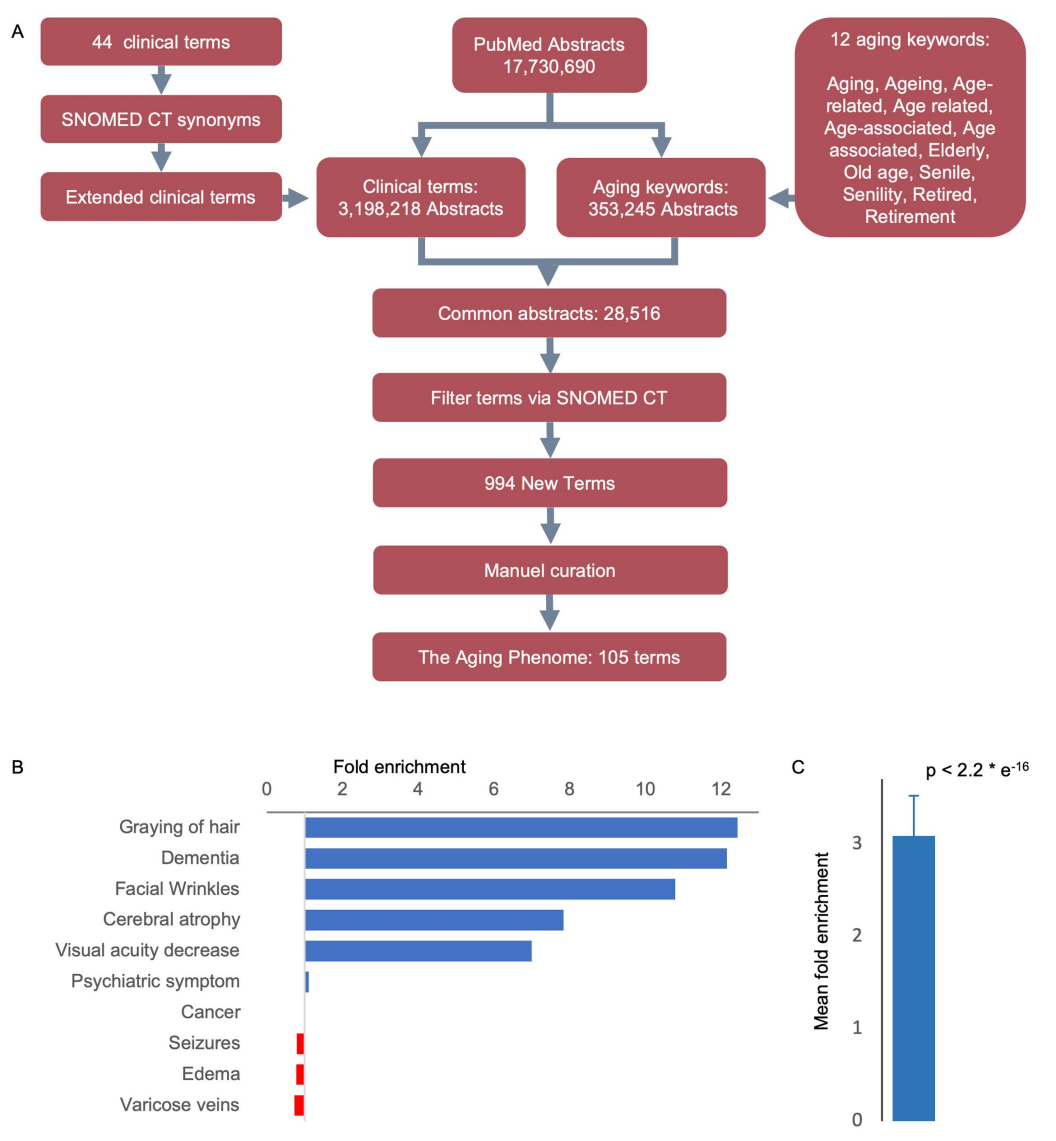
**An approach to identifying age-related features.** (**A**) Workflow-diagram of the project. (**B**) Top and bottom clinical terms that are enriched in the aging dataset (see Figure S1 for the expanded list). (**C**) Mean enrichment of the terms (Mean ± SEM, n = 44, p-value determine by Chi-square test, see Figure S2 for individual terms).

As a starting point for defining human aging we used 44 clinical terms that we had previously used to describe human aging [7–9]. To increase our ability to capture semantically similar age-associated terms we extracted synonyms and spelling analogues for each of these 44 clinical terms from the SNOMED CT terminology, which contains a comprehensive and validated collection of terms describing clinical features (Table S1, Figure S1A) [10]. In all subsequent analyses using the 44 clinical terms we also included their synonyms and spelling analogues. To quantitatively test whether the terms in the list are associated with human aging, we measured their enrichment in aging-related abstracts when compared to all PubMed abstracts. To that end, we mined 17,730,690 PubMed abstracts for occurrences of the 44 clinical terms and investigated whether they co-occur with the word aging. In addition to aging we included other ‘aging keywords’ with similar semantic meaning, e.g., elderly, old age, retirement (Figure 1A and Table S2, Figure S1B). Indeed, the 44 terms were enriched 3.1-fold (mean, p-value < 2e^-16^, chi-squared test) in abstracts that also contained aging keywords, suggesting that this list could be used as bait for finding other terms describing aging (Figure 1B, 1C and Figure S2).

To qualitatively test the algorithms’ ability to find new terms, we selected 100 random abstracts and manually picked out terms of interest to determine if the text-mining algorithm would be able to capture them. We then calculated the F-measure (F1 score) based on the precision and recall of the algorithm [11]. This score is determined by identifying how many terms are included and how many are missing in the abstracts by comparing a manual selection versus the automated algorithm. The algorithm was calculated to have an Fl score of 0.898, suggesting that our text-mining algorithm captures the majority of terms allowing us to interrogate the aging phenotype.

### Mining for potential aging-associated phenotype terms

We next identified 3,198,218 PubMed abstracts containing one or more of the 44 age-associated clinical terms and 431,949 abstracts containing two or more of the 44 age-associated clinical terms. We speculated that abstracts containing two or more age-associated clinical terms are more accurately associated with aging compared to abstracts containing just one term. For example, if we search for abstracts containing the single term ‘cancer’ we would possibly find terms that show only minor association with aging. We therefore compared the frequency of co-occurrence of each of the terms by dividing the number of times a term is mentioned together with any other term versus when it is mentioned on its own (Figure S3). Indeed, if we only considered abstracts where single clinical-terms were mentioned we observed that very common terms, like ‘cancer’, skewed the entire dataset towards those terms instead of aging. We therefore only considered abstracts that contain two or more age-associated clinical terms for finding new terms that describe human aging.

Employing this approach, we identified 28,516 PubMed abstracts which contain: 1) at least two occurrences of the 44 clinical terms, and 2) at least one aging keyword. These age-associated abstracts were then used as a foundation for mining new terms associated with aging. We generated a list of the most frequent words in the age-associated abstracts. We chose a cutoff of at least 100 occurrences, including repeated occurrences of a term in an abstract, as a way to filter the number of terms identified and to make sure that only well-recognized terms are included. We discarded terms based on their semantic tags in SNOMED (e.g., “procedure”, “qualifier value”, “body structure”). This led to the identification of 994 new terms that could be considered age-associated (Table S3).

### Association analyses reveal tissue specific clustering in aging

**Figure 2:**
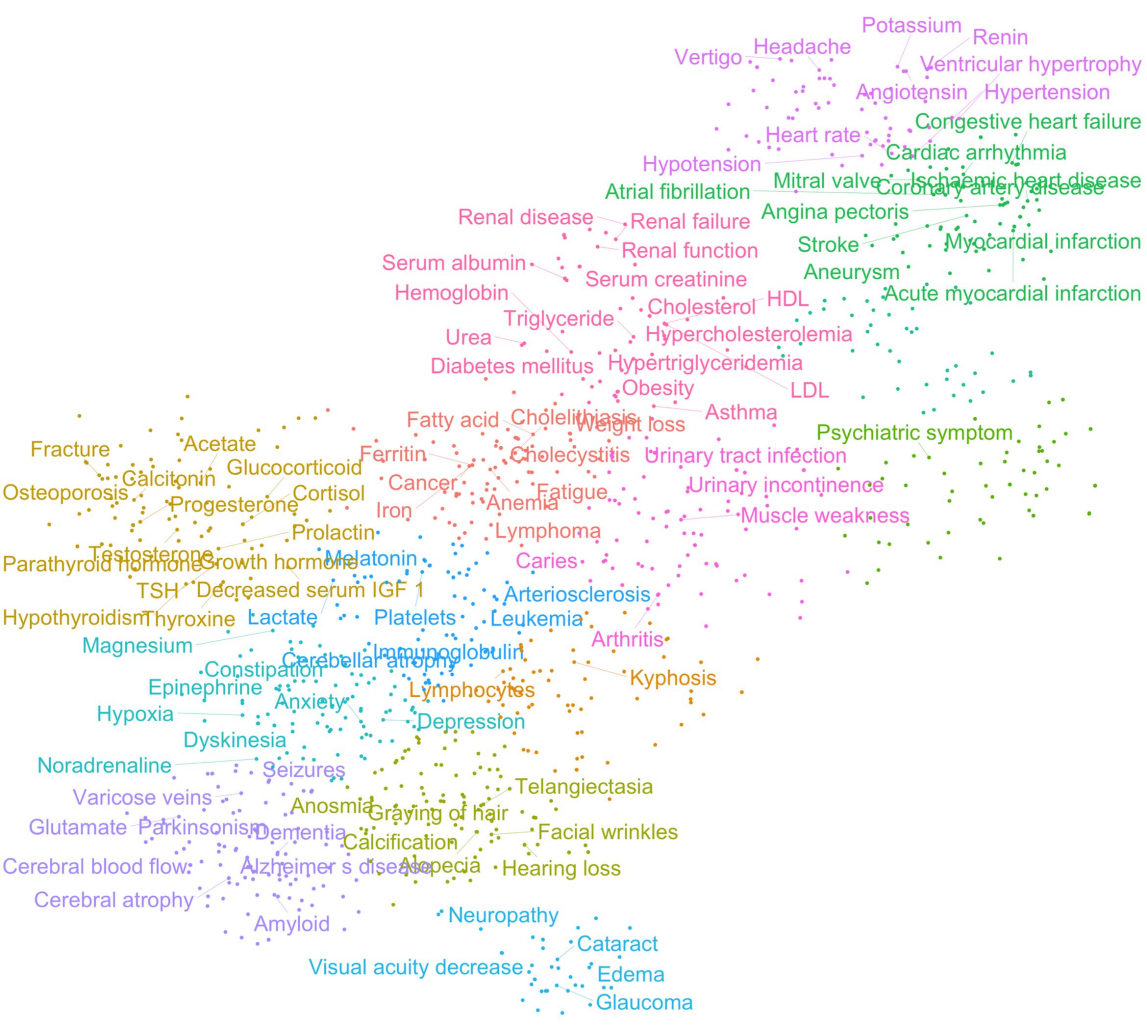
**Age-associated clinical terms show distinct pathological clusters.** T-distributed Stochastic Neighbor Embedding (t-SNE) clustering of z-score normalized data.

To further investigate the relationships between these features, we generated a clinical term matrix reflecting the co-occurrence of terms in each abstract. To avoid bias towards terms that were more commonly or less commonly mentioned than average, we employed both standard score (z-score) and term frequency–inverse document frequency (tf-idf) normalization [12,13]. These two normalization algorithms compensate for the ways in which terms associate differently: z-score emphasizes connections between more rare co-occurrences while tf-idf emphasizes correlations between more common terms. By using these matrices, we could perform further analyses and investigate how different features associate with each other. To find large-scale patterns in the data we applied T-distributed Stochastic Neighbor Embedding (t-SNE) clustering to the matrices. This unsupervised machine-learning algorithm allowed us to identify groups of terms that appeared closely associated (Figure 2 and Figure S4). In particular, it was apparent that terms relating to specific pathologies (e.g., heart disease, neurodegeneration) associate with one another, thereby validating our normalization methods. Notably, the term cancer appeared to associate with a cluster including ‘iron’, *‘*Ferritin’, ‘Anemia’ suggesting that these are possible markers for cancer identification/progression. Indeed, this may be the case [14]. In sum, these algorithms show that the results generated from our data-mining effort agree with current knowledge and suggest that our method is robust.

**Figure 3:**
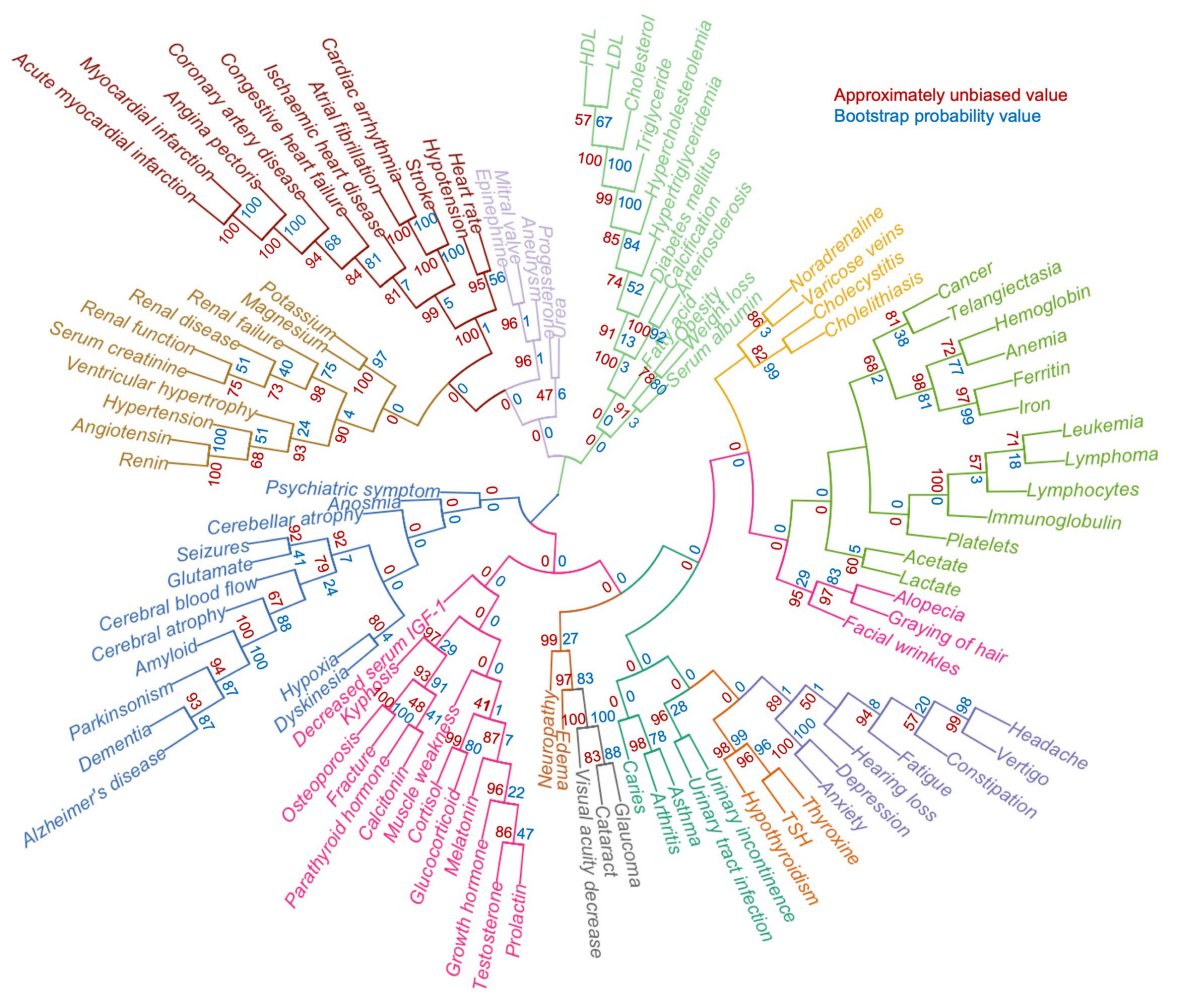
**A defined aging phenome shows functional clustering.** Agglomerative hierarchical clustering of 105 clinical terms describing human aging based on z-score normalized representation in the literature. Colors represent different clusters. The approximately unbiased value is shown in red while the bootstrap probability value is shown in blue.

While the 994 terms represent an unbiased list of age-associated terms, it was apparent that many terms are not descriptive of the aging phenotype. To further condense the list of features, we manually curated the list, allowing us to identify 105 age-associated terms that could constitute the aging phenome (Table S4). To understand how these terms correlate with each other, we performed agglomerative hierarchical clustering analysis and created heatmaps of the co-occurrence of the terms. Notably, this allowed us to identify features that are co-associated with each other in aging (Figure 3 and Figure S5A-C). While t-SNE clustering appeared to work well with both tf-idf and z-score normalized data, hierarchical clustering only gave good and meaningful separation using z-score normalized data. Indeed, using this normalization, broad clusters were apparent representing major organ systems. For example, musculoskeletal terms formed a separate cluster, neurological terms another, etc. A number of interesting observations were evident from the clustering. For instance, kidney function appeared to associate more closely with cardiovascular disease than the metabolic cluster containing cholesterol; and facial wrinkles, alopecia and graying of hair associated with a hematological aging cluster.

**Figure 4:**
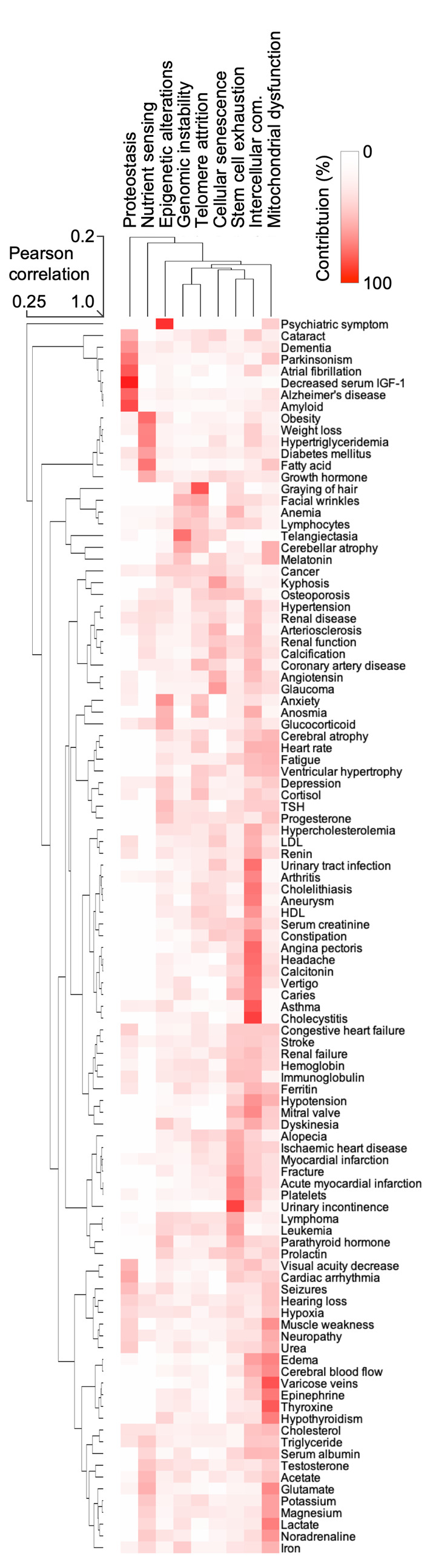
**The hallmarks of aging are associated with certain human features.** Heatmap and cluster analysis of the association between age-associated clinical terms and hallmarks of aging.

Nine cellular and physiological hallmarks have been associated with aging [1]. To understand how each hallmark might contribute to the aging phenome and the observed clustering of terms, we mined the PubMed data for the hallmark terms and their synonyms (Table S5) allowing us to rank how each hallmark contributes to each term. This allowed us to generate a hierarchical clustering and heatmap of the terms and their relationships with the hallmarks (Figure 4). Quite strikingly, clusters of terms were associated with specific hallmarks, suggesting that these hallmarks are driving that specific cluster. For example, neurodegenerative diseases were associated with the proteostasis hallmark, while a metabolic cluster of obesity, weight loss, hypertriglyceridemia was associated with the nutrient sensing aging hallmark. This approach also allowed us to understand how the hallmarks relate to each other. It was evident that genomic instability was associated with telomere attrition; and stem cell exhaustion appeared to be associated with altered intercellular communication. These two clusters were associated with cellular senescence. While this approach gives us a good understanding of how the different terms associate with each other and the potential underlying molecular basis of this clustering, it remains unclear how each term contributes to aging.

It is likely that not all features are equally important in aging. We therefore weighed the terms based on the frequency of their occurrence in abstracts also containing the aging keywords. We found 170,350 abstracts containing the 105 terms from the final list and aging keywords. Terms were counted as present or absent with a frequency ranging from 10 to 26,845 abstract occurrences (mean of 2702.67, Figure S6). Notably, we found dementia, cancer, depression, and hypertension among the most strongly age-associated terms in the literature. Interestingly, terms like “graying of hair” and “facial wrinkles” were among the 10 least frequent terms, despite being highly prevalent in the aging population [15]. This indicates a discrepancy between the perceived importance in PubMed and the real prevalence in the general population.

### Manual curation producing a weighted aging phenome

**Figure 5:**
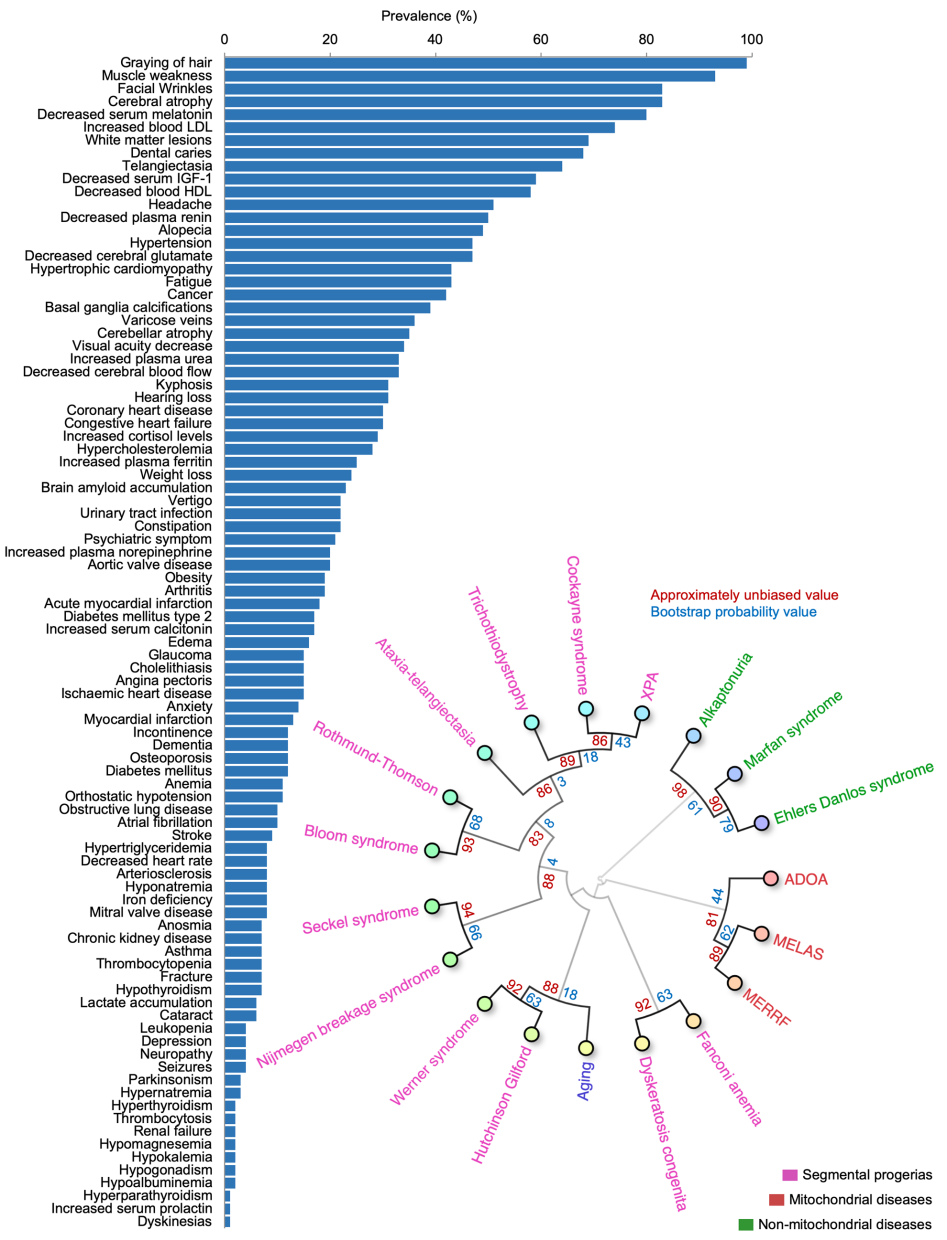
**The aging phenome.** (**A**) The prevalence of features in the elderly (manually curated literature describing 76,928,696 individuals). HDL: High density lipoprotein, IGF-1: Insulin like growth factor-1, LDL: Low density lipoprotein (**B**) Agglomerative hierarchical clustering using uncentered similarity and average linkage of aging and genetic diseases (red: primary mitochondrial disorders, green: non-mitochondrial disorders, purple: segmental progerias). The approximately unbiased value is shown in red while the bootstrap probability value is shown in blue. ADOA: Autosomal dominant optic atrophy, MELAS: Mitochondrial encephalopathy, lactic acidosis, and stroke-like episodes, MERRF: Myoclonic epilepsy with ragged-red fibers, XPA: Xeroderma pigmentosum complementation group A.

To understand the importance of these terms in normal aging, we manually identified papers describing cohorts of individuals where the prevalence of the new age-associated terms was described. Since some features reflect similar processes (e.g., increased serum levels of creatinine and kidney disease), these terms were combined to allow subsequent comparative analyses. In total, the prevalence of the features from a variety of published cohorts describing a total of 76,928,696 individuals aged 65 years or older where identified, allowing us to comprehensively describe the prevalence of the different features in aging (Figure 5A) [15–70]. This process further allowed us to compare how human aging is associated with other diseases based on the prevalence of features [7–9]. To this end, we performed hierarchical clustering between aging and known premature aging diseases, primary mitochondrial disorders, and some non-mitochondrial control diseases [71]. Notably, aging clustered strongly with known premature aging diseases: Werner syndrome and Hutchinson-Gilford progeria, and these in turn clustered with primary mitochondrial diseases (Figure 5B). In sum, we were able to define and quantify a human aging phenome covering all tissues in the body.

## DISCUSSION

Aging is among the most complex phenotype in humans. Indeed, the list of features found here reflects the multitude of pathologies associated with aging. Of note, we were able to identify a number of large-scale clusters within the aging phenome that associate with specific molecular pathways. This finding could indicate that clustered phenotypes share common etiologies. Indeed, loss of proteostasis is associated with multiple neurodegenerative diseases and this appeared to be corroborated with our approach. Quite interestingly, this could also indicate that there are only a few underlying processes driving each of the different phenotype clusters, suggesting that targeting these root causes may be a good strategy for treating multiple age-associated pathologies.

Determining the importance of the different features of aging is challenging. Herein, we created a ranked list of features associated with aging. The top ranked term was dementia followed by cancer, depression, hypertension, fractures, and stroke. Notably, while these features are certainly important, they are much less prevalent than features such as muscle weakness, facial wrinkles and graying of hair. Indeed, such features were considerably underrepresented in the aging research literature despite being some of the most prevalent in human aging. One could speculate that these under-represented features of aging could be good research targets in future studies. Further, the fact that some features are highly prevalent yet rarely studied could indicate that our data-mining approach incompletely describes human aging. Nevertheless, the identified terms appear to describe human aging comprehensively as well as have clinical relevance in terms of premature aging diseases. Further, the terms cluster well with each other and with the previously manually generated list of 44 terms indicating that our approach appears to be valid.

In conclusion, the aging phenome could be used in a myriad of applications. For instance, the critical knowledge of the aging phenome could determine possible outcomes for clinical trials, for identifying new biomarkers of aging and for discovering how different pathologies arise in aging. As shown, the aging phenome could also be used to better classify premature aging diseases, a group of disorders that could be of interest in understanding the mechanistic basis of aging.

## MATERIALS AND METHODS

### Software

All source code used in this paper can be found at https://github.com/scheibye-knudsen-lab/aging-phenome/ .

### PubMed Baseline Repository

17,730,690 journal abstracts from the PubMed Baseline Repository [72] (Last Updated November 28, 2017) were downloaded and used for subsequent analyses.

### SNOMED clinical terms

SNOMED CT [10] was used as a source of terms as well as synonyms and spelling analogues for terms.

### Synonyms of age-associated clinical terms

44 previously described age-associated clinical terms [7] were augmented with up to 20 synonyms and spelling analogues as defined in SNOMED CT [73], e.g. “Graying of hair”, “Gray Hair” (Table S1). Synonyms and spelling analogues were counted towards their corresponding original term.

### Aging keywords

To identify abstracts that are associated with aging, we used a list of aging keywords, e.g. “aging”, “aging related”, “old age” and “retirement” along with spelling analogues, e.g. “ageing”, “ageing-related” (Table S2).

### Abstract identification and word matrix generation

PubMed abstracts were searched for the presence of the 44 age-associated clinical terms yielding a feature matrix signifying the presence or absence of each of the terms in each of the 17,730,690 PubMed abstracts. We then discarded abstracts where no age-associated clinical terms were present yielding a remaining 3,198,218 abstracts with one or more terms present. Similarly, we constructed a matrix signifying the presence or absence of each of the 12 aging keywords in the 17,730,690 PubMed abstracts. 353,245 abstracts were found to contain one or more of the 12 aging keywords. 28,516 abstracts were identified where both an aging-keyword and at least two clinical terms were present and this was used to generate a matrix of combined terms.

### Precision and recall (F1 score)

The precision of the search algorithm to find all the correct clinical terms was examined by selecting 100 random abstracts and manually identifying terms of interest. Hereafter, the terms were counted if found (or not) by the search algorithm. To evaluate the precision of the algorithm we calculated an F-measure (F1 score) for the terms that were found (true positive) and that were not found (false negative) by the algorithm, compared with the manually identified terms.

### Enrichment of age-associated clinical terms in abstracts containing aging keywords

The total number of times a clinical term was present in the 353,245 abstracts containing aging keywords was calculated. This is the aging-count. To find the expected count, 353,245 abstracts were randomly selected from the entire data set of 17,730,690 abstracts and the total number of times a clinical term was present was calculated. This was repeated 100 times and the average total count per clinical term was calculated. This is the expected-count. The ratio between the aging-count and the expected-count was then calculated per clinical term as a measurement of enrichment of terms in age-associated abstracts.

### New aging clinical terms from PubMed abstracts

67,901 SNOMED CT terms were found to be present in the 28,516 abstracts where both an aging-keyword and at least two clinical terms were present (see above). We discarded terms mentioned less than 100 times as those would be weaker candidates for newly discovered aging clinical terms. This reduced the list of potential new terms to 10,486.

This list of terms was filtered based on the following unwanted semantic tags associated with SNOMED CT terms: ‘procedure’, ‘qualifier value’, ‘body structure’, ‘attribute’, ‘organism’, ‘person’, ‘regime/therapy’, ‘ethnic group’, ‘environment’, ‘physical object’, ‘tumor staging’, and ‘geographic location’. Words containing ‘/’ signified unit measures and were also removed. This resulted in a final list of 994 terms.

### Word matrix normalization

A combined set (n=1050) of age-associated clinical terms (44) and aging keywords (12) as well as the newly generated list of clinical terms (994) was projected into a count-matrix that consisted of 1050 terms against 28,516 abstracts. The value inserted in the matrix was 1 if the term was present in the abstract and 0 if it was absent. For normalization the values were subsequently converted into (1) z-score (z-score_x_ = (mean – value_x_) / standard deviation) using the python scipy.stats.zscore library and (2) tf-idf using the sklearn.feature_extraction library [74].

### Manual curation of new age-associated clinical terms

We manually curated the list of 994 candidate age-associated clinical terms to exclude concepts that are clearly not aging related, e.g. “disorder” or “enzyme”, yielding a list of 105 terms: the aging-phenome.

### Hallmarks of aging analysis

The nine hallmarks of aging were augmented with up to three synonyms from SNOMED CT. The 17,730,690 abstracts were then mined for mentions of one or more of the nine hallmarks of aging. The abstracts containing hallmarks of aging (673,409) were then mined for the 105 human aging-phenome terms. First, we counted the co-occurrence of each of the 105 aging phenome terms with each hallmark of aging, and summarized in a count matrix with terms as rows and hallmarks as columns. Second, the count matrix was normalized, by dividing each value in the matrix by the total count (sum of the column) of each hallmark of aging. Third, for each term, we calculated the percentage contribution from each hallmark of aging. This percentage matrix was used for the generation of a heatmap and agglomerative hierarchical clustering.

### Circular dendrogram

Agglomerative hierarchical clustering of the z-score and tf-idf normalized matrices was performed using Euclidian distance and average similarity, and plotted in a circular dendrogram using the ‘ape’ R package. We manually identified 14 clusters of terms that fit well together, e.g. Glaucoma, Cataract and Visual acuity decrease, and colored the dendrogram accordingly.

### T-distributed Stochastic Neighbor Embedding (t-SNE)

T-distributed Stochastic Neighbor Embedding (t-SNE) [75] was performed using TensorFlow’s online implementation of t-SNE (projector.tensorflow.org) by loading the matrix of the 105 word-vectors on 28,516 PubMed abstracts. Since we applied normalization to the data, we turned TensorFlow’s spherize data feature off. We elected to visualize the results in two-dimensional space and accepted TensorFlow’s default values for perplexity (=9) and learning rate (=10.) The algorithm was allowed to run for 10,925 iterations. We then applied k-means clustering to the two-dimensional t-SNE coordinates and colored the clusters accordingly. We elected to cluster 14 centers in accordance with the number of clusters we identified manually in the circular dendrogram.

### Heatmap

We used the ‘Pheatmap’ R package [76] to generate a clustered heatmap of the z-score and tf-idf normalized matrices of the 105 aging phenome. Agglomerative hierarchical clustering for both the terms and abstracts was done using average-linkage and Euclidian distance.

### Term frequency in abstracts

We performed a text search for the 105 terms with synonyms to evaluate the occurrence frequency of these terms in the literature. The analysis was performed only on the 353,245 abstracts also containing aging keywords. The terms with synonyms were collapsed into the ‘main’ term and only counted as present (one) or absent (zero) in each abstract.

### Identification of prevalence

Manual identification of the prevalence of each of the 105 terms in populations aged 65 or older was performed by searching PubMed for articles where cohorts were described. Each term was searched in PubMed along with the keywords ‘prevalence’ and/or 'clinical’ and/or ‘elderly’/’aging’/’retired’. For each term we attempted to identify the most recent and largest cohort available. In some cases we had to calculate how many elderly individuals had abnormal values compared to young individuals. For example, average lactate increases with age [16], but to define a prevalence of “lactate accumulation” we calculated the percentage of elderly individuals that were more than 2 standard deviations different from young individuals. In some cases, terms were redefined to more descriptive terms. For example, the term “platelets” was redefined to “thrombocytopenia” and “thrombocytosis,” and the prevalence of those terms was identified.

### Statistics

Statistical tests were conducted as indicated in the text. Bootstrap resampling (100 iterations) was applied to hierarchical clustering using the ‘pvclust’ R package [77].

## Supplementary Material

Supplementary Figures

Supplementary Tables
