# Supplementary material for: A defined human aging phenome
Source: Aging (Albany NY). 2019 Aug 12;11(15):5786–806. doi: 10.18632/aging.102166 (PMC11627290; doi:10.18632/aging.102166)
Supplement: Supplementary Figures [file aging-11-102166-s001.pdf]

SUPPLEMENTARY FIGURES

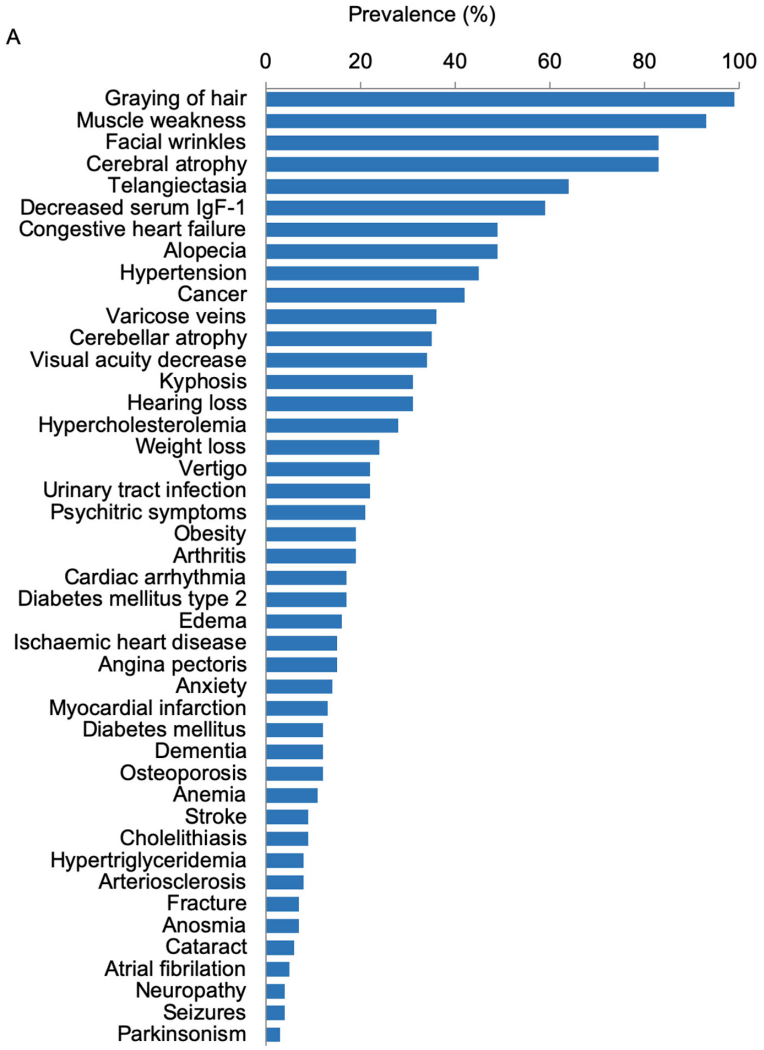

Figure S1. Age-associated clinical terms and the PubMed repository. (**A**) 44 age-associated clinical terms and their prevalence in the elderly population. (**B**) Distribution of PubMed abstracts used in the analyses according to their publication date.

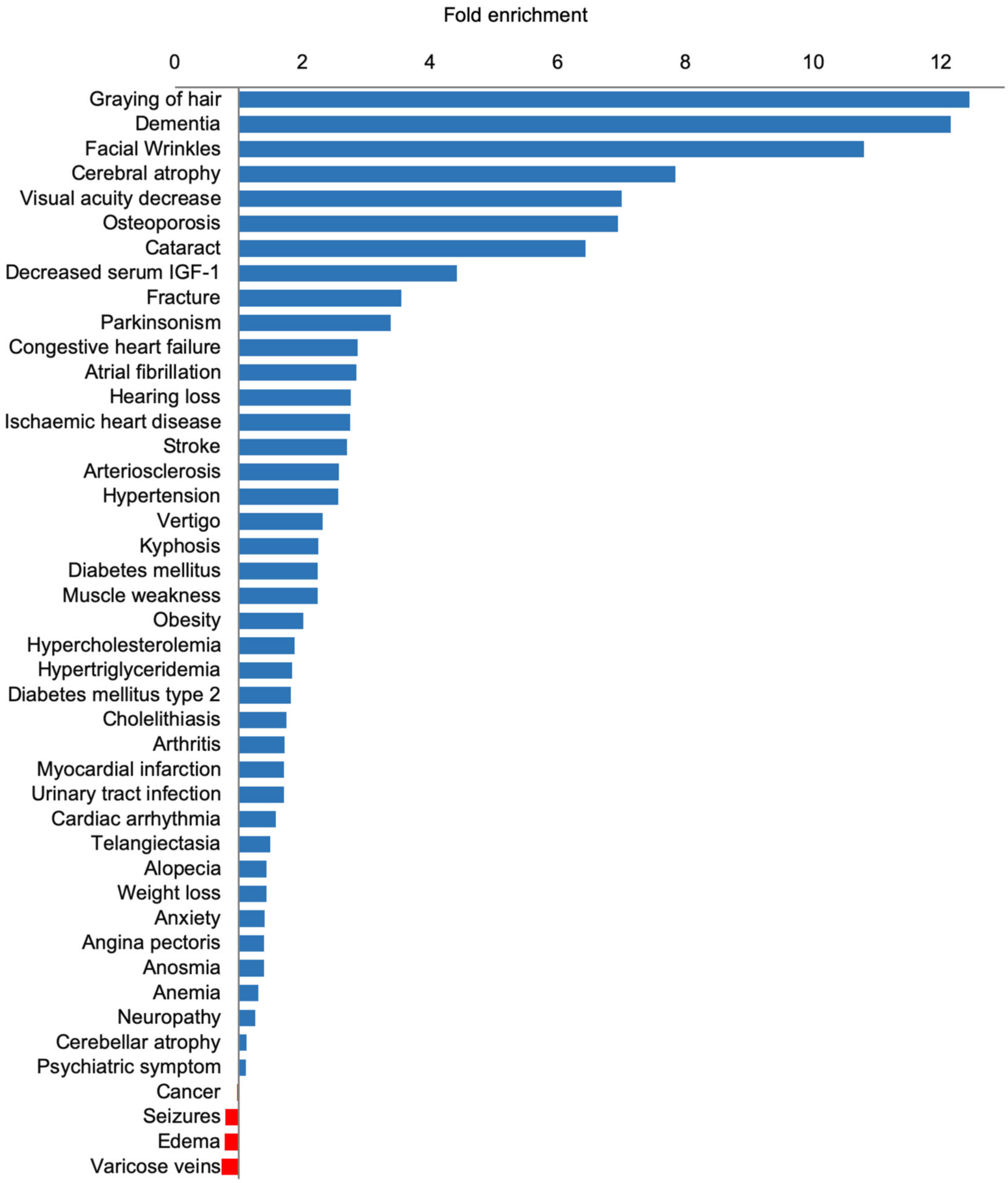

Figure S2. Fold enrichment of the 44 clinical terms in abstracts containing aging keywords. The fold enrichment of the 44 clinical terms in abstracts containing aging keywords compared to a calculated expected number of terms occurring in 100 randomly sampled abstracts.

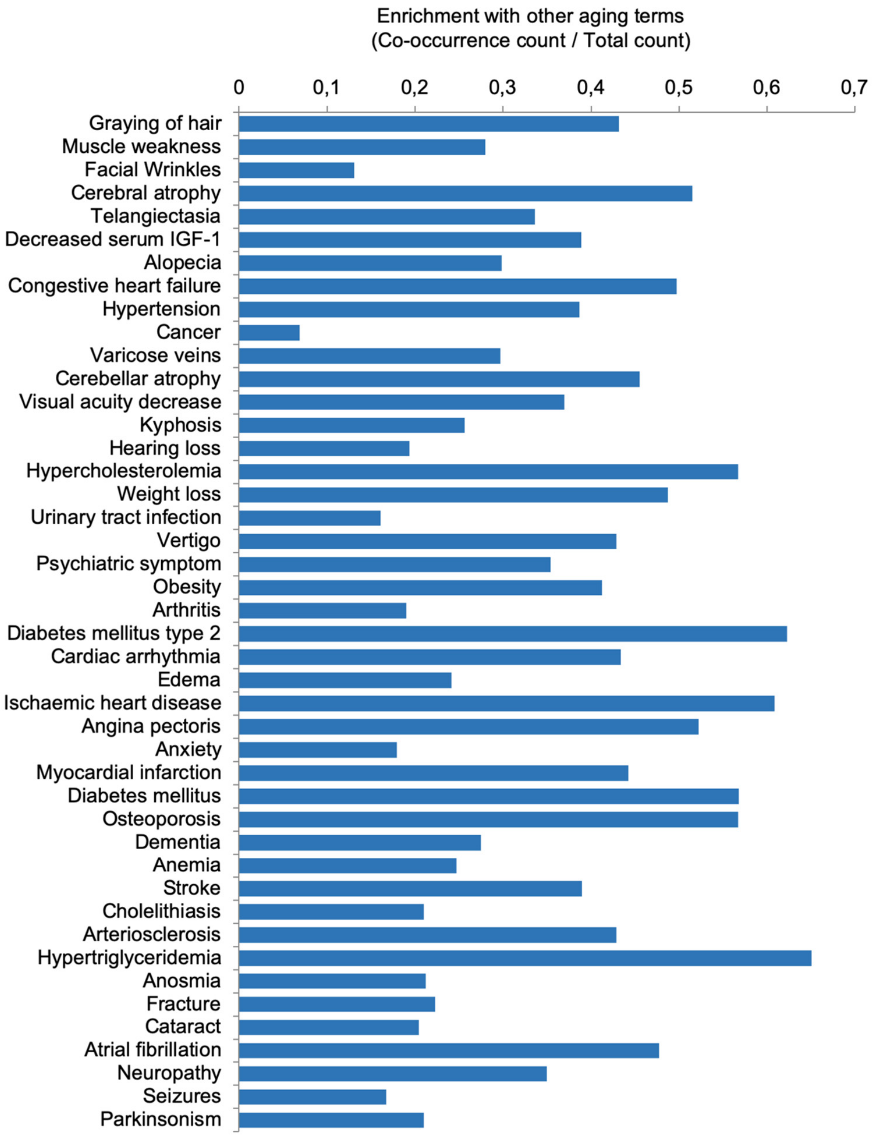

Figure S3. Including two clinical terms as search bait leads to less data skewing. Graph shows fold difference in the identified terms count using abstracts with at least one of the 44 clinical terms versus using abstracts with co-occurrence of the 44 clinical terms.

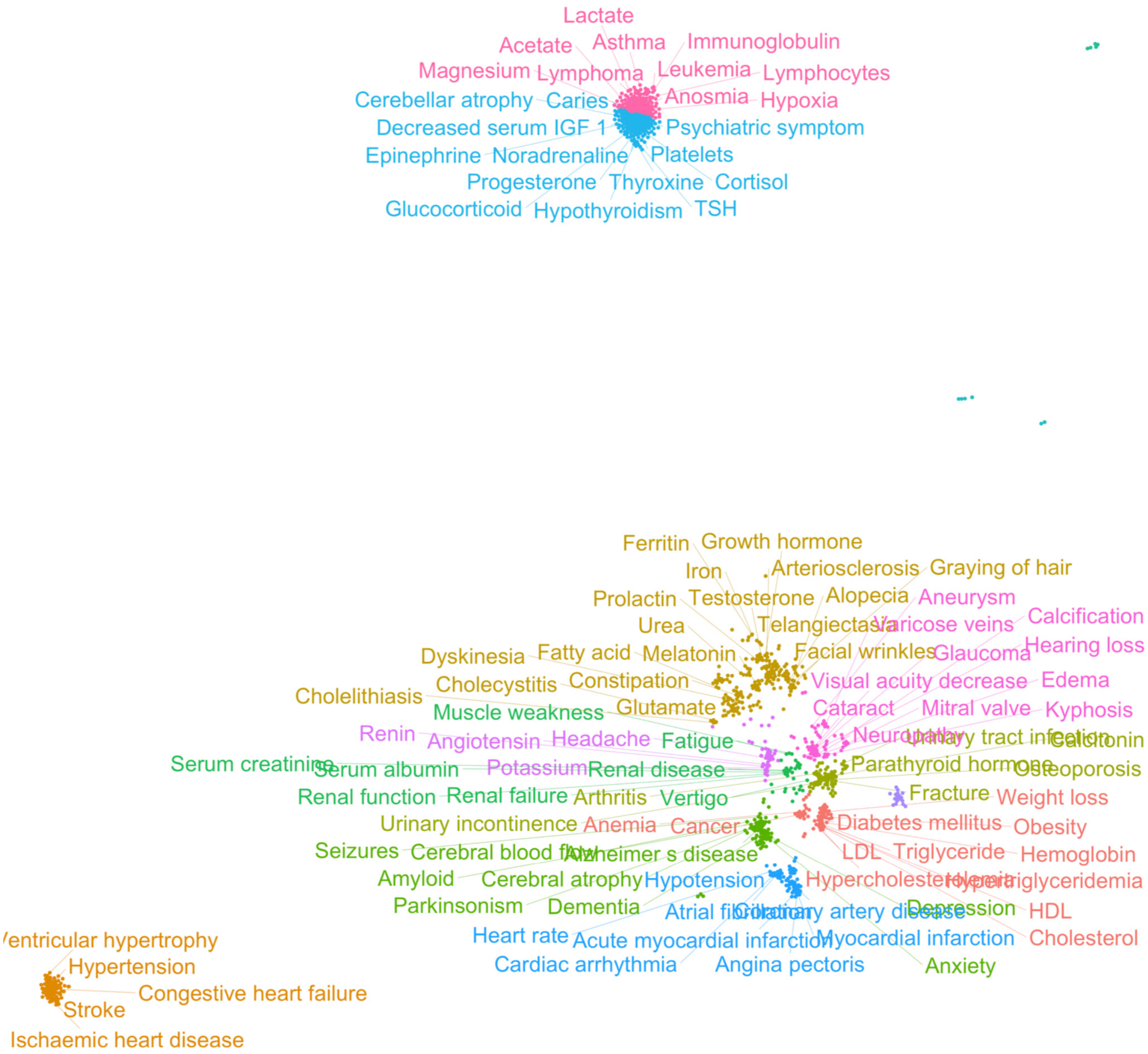

Figure S4. Machine-learned clustering of age-associated terms. t-SNE clustering of term frequency–inverse document frequency (tf-idf) normalized data. Coloration is based on k-means clustering (14 clusters) of the 2D data.

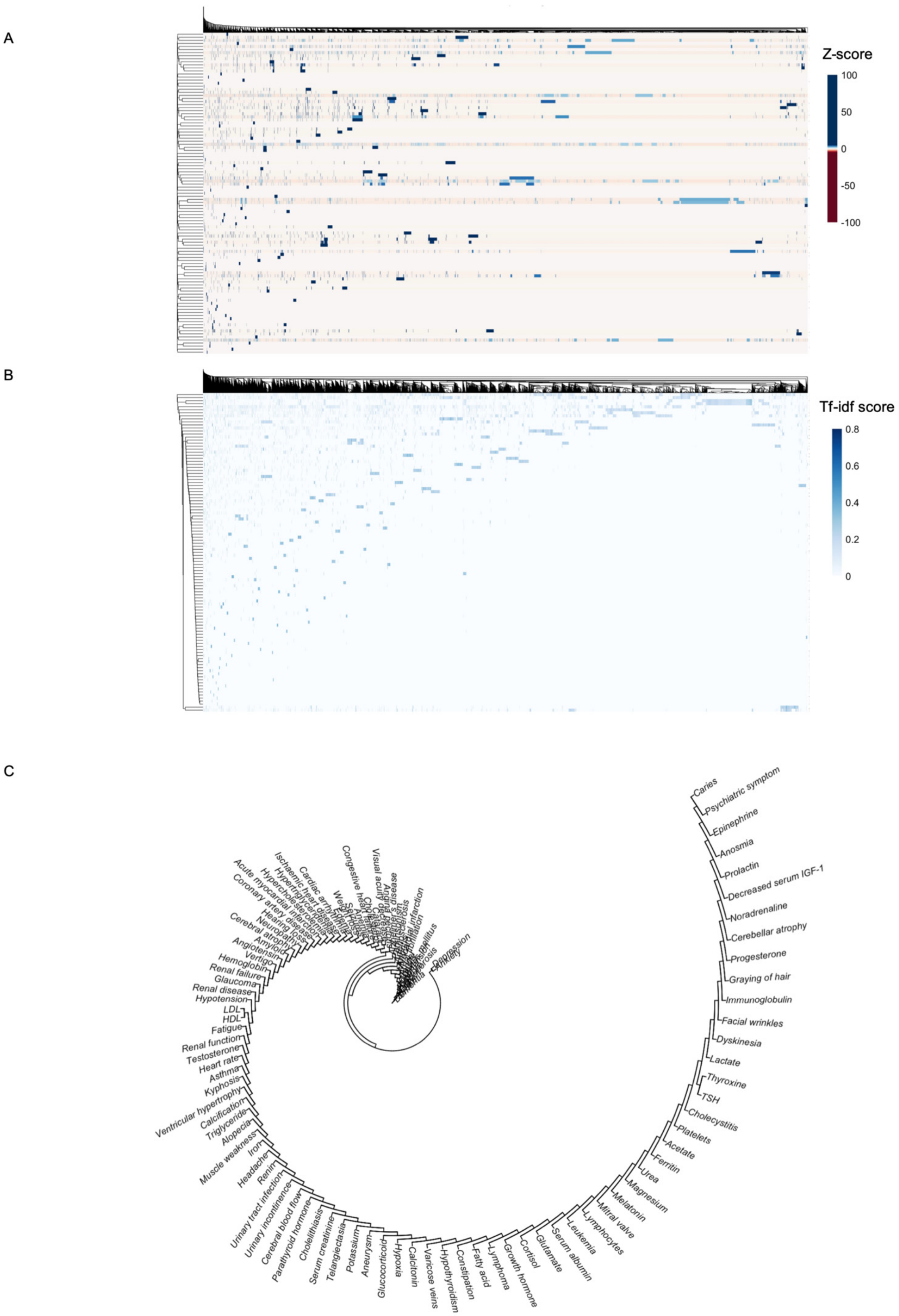

Figure S5. Distinct clusters can be identified in z-score or tf-idf normalized data. (**A**) Heatmap of z-score normalized data. (**B**) Heatmap of tf-idf normalized data. C Agglomerative hierarchical clustering of tf-idf normalized data.

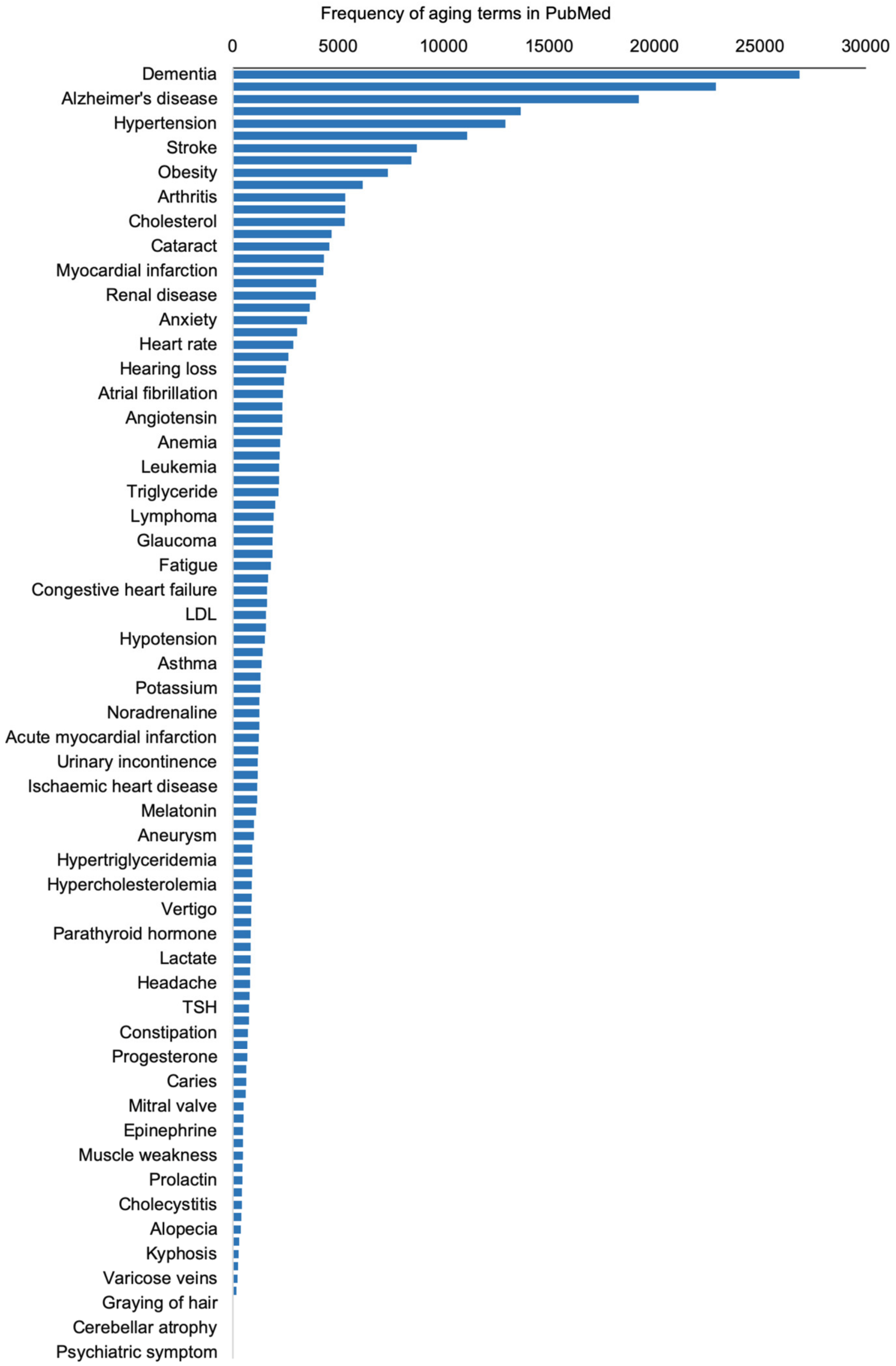

Figure S6. The 105 age-associated clinical terms occurrence frequency in PubMed abstracts containing the aging synonyms. The frequency of occurrence of the 105 age-associated clinical terms in abstracts also containing aging synonyms.
