# Supplementary material for: A defined human aging phenome
Source: Aging (Albany NY). 2019 Aug 12;11(15):5786–806. doi: 10.18632/aging.102166 (PMC11627290; doi:10.18632/aging.102166)
Supplement: Supplementary Tables [file aging-11-102166-s002.pdf]

# SUPPLEMENTARY TABLES

## Table S1. List of terms describing clinical features.

1. Graying of hair
   - Gray Hair
   - Greying of Hair
   - Grey hair
   - Canities
2. Muscle weakness
   - Weakness of muscle
   - Weakness of the muscle
   - Musculoskeletal weakne
   - Myoskeletal weakness
   - Muscle strength reducec
   - Decreased muscle streng
3. Facial Wrinkles
   - Wrinkled facies
   - Skin wrinkles
   - Wrinkled skin
   - Lined face
   - Wrinkled face
   - Perioral wrinkles
   - Perioral rhytides
   - Rhytid
   - Rhytides
4. Cerebral atrophy
   - Atrophy of the cerebrun
   - Brain atrophy
   - Atrophy of the cerebrun
   - Atrophy of brain
5. Telangiectasia
   - Telangiectasis
6. Decreased serum IGF-1
   - Decreased IGF-1
   - Low serum IGF-1
   - Low IGF-1
   - Decreased insulin like growth factor 1
   - Decreased serum insulin like growth factor 1
   - Low insulin like growth factor 1
   - Low serum insulin like growth factor 1
   - Decreased serum IGF 1;Decreased IGF 1
   - Low serum IGF 1
   - Low IGF 1
   - Decreased insulin-like growth factor I
   - Decreased serum insulin-like growth factor I
   - Low insulin-like growth factor I
   - Low serum insulin-like growth factor I
   - Decreased insulin like growth factor I
   - Decreased serum insulin like growth factor I
   - Low insulin like growth factor I
   - Low serum insulin like growth factor I
7. Alopecia
   - Hair loss
   - Loss of hair
   - Baldness
   - Bald
   - Falling hair
   - Thinning hair
8. Congestive heart failure
   - Congestive heart disease
   - Congestive cardiac failure
9. Hypertension
   - High blood pressure
   - Increased blood pressure
   - Hypertensive disorder
   - Hypertensive vascular disease
   - Elevated blood pressure
   - Raised blood pressure
   - Blood pressure elevation
10. Cancer
    - Malignacy
    - Malignancy
    - Neoplasm, malignant
    - Unclassified tumor, malignant
    - Tumour, malignant
    - Tumor, malignant
    - Unclassified tumour, malignant
    - Malignant neoplasm
    - Neoplasm, malignant
    - Malignant tumor
11. Varicose veins
    - Venous varices
    - Varices
    - Varicosities
12. Cerebellar atrophy
    - Atrophy of the cerebellum
13. Visual acuity decrease
    - Loss of vision
    - Vision loss
    - Visual impairment
    - Impaired vision
    - Visual difficulty
14. Kyphosis
    - Increased spinal curvature
    - humpback
    - Gibbosity
    - Gibbus
    - Hunchback
    - Deformity of spine
15. Hearing loss
    - Hearing impairment
    - Loss of hearing
    - Deafness
    - Impaired hearing
    - Hard of hearing
    - Difficulty hearing
    - Hypoacusis
    - Presbycusis
    - Senile deafness
16. Hypercholesterolemia
    - Hypercholesterolaemia
    - High cholesterol
17. Weight loss
    - loss of bodyweight
18. Urinary tract infection
    - Urinary tract infectious disease
19. Vertigo
    - Dizziness
20. Psychiatric symptom
21. Obesity
    - Fatness
    - Overweight
    - Adiposis
    - Adiposity
22. Arthritis
    - Osteoarthritis
    - Arthrosis
    - Joint inflammation
    - Degenerative joint disease
    - Degenerative arthropathy
    - Osteoarthrosis
    - Arthropathy
23. Diabetes mellitus type 2
    - Diabetes 2
    - Type 2 Diabetes
    - Diabetes mellitus Type II
    - Diabetes Type 2
    - Diabetes Type II
24. Cardiac arrhythmia
    - Heart arrhythmia
    - Arrhythmia of the heart
    - Arrhythmia
    - Cardiac dysrhythmia
    - Disorder of heart rhythm
25. Edema
    - Swelling
    - Oedema
    - Oedematous
    - [illegible]
26. Ischaemic heart disease
    - Ischemic heart disease
27. Angina pectoris
    - Chest pain
    - Angina
    - Cardiac angina
    - Angina pectoris
    - Stenocardia
    - Anginal syndrome
28. Anxiety
    - Anxiousness
    - Feeling anxious
29. Myocardial infarction
    - Infarction of heart
    - Cardiac infarction
    - Heart attack
    - Myocardial infarct
30. Diabetes mellitus
31. Osteoporosis
32. Dementia
    - Cognitive impairment
    - Impaired cognition
    - Cognitive disturbance
    - Cognitive dysfunction
    - Cognitive decline
    - Cognitive deficit
33. Anemia
    - Low hemoglobin
    - Decreased hemoglobin
    - Low erythrocytes
    - Decreased erythrocytes
    - Anaemia
    - Hemoglobin low
    - Haemoglobin low
34. Stroke
    - Apoplexy
    - Apoplexia
    - Cerebrovascular accident
35. Cholelithiasis
    - Gallstone
    - Biliary calculus
    - Cholelithiasis
    - Stone - biliary
    - Calculus - biliary
    - Calculus in biliary tract
    - Gallstones
    - Gallbladder stones
    - Gallbladder calculus
36. Arteriosclerosis
    - atherosclerosis
37. Hypertriglyceridemia
    - Hyperlipidemia
    - Hypertriglyceridaemia
    - Lipidemia
    - Lipidaemia
    - Hyperlipidaemia
38. Anosmia
    - Loss of smell
    - Loss of sense of smell
    - Loss of the sense of smell
    - Absent smell
    - No sense of smell
    - Sense of smell lost
    - Sense of smell absent
39. Fracture
    - Fractures
    - Broken bones
    - Broken bone
40. Cataract
    - Cataracts
    - Opaqueness of the lens
    - Lens opaqueness
    - Lens opacity
    - Lens opacities
    - Lenticular opacity
41. Atrial fibrillation
42. Neuropathy
43. Seizures
    - Seizure
    - Epilepsy
    - Convulsion
    - Epileptic
44. Parkinsonism
    - Parkinsons dise
    - Parkinson's dis
    - Paralysis agitar
    - Shaking palsy
    - Parkinson disea

**Table S2. List of 'aging' keywords.**

Aging
Ageing
Age-related
Age related
Age-associated
Age associated
Elderly
Old age
Senile
Senility
Retired
Retirement

**Table S3. List of terms that were mined from PubMed based on the aging keywords (marked in yellow) and the clinical terms (marked in green).**

Ageing
Aging
Age-related
Age related
Age-associated
Age associated
Elderly
Old age
Senile
Senility
Retired
Retirement
Graying of hair
Muscle weakness
Facial wrinkles
Cerebral atrophy
Telangiectasia
Decreased serum IGF-1
Alopecia
Congestive heart failure
Hypertension
Cancer
Varicose veins
Cerebellar atrophy
Visual acuity decrease
Kyphosis
Hearing loss
Hypercholesterolemia
Weight loss
Urinary tract infection
Vertigo
Psychiatric symptom
Obesity
Arthritis
Diabetes mellitus type 2
Cardiac arrhythmia
Edema
Ischaemic heart disease
Angina pectoris
Anxiety
Myocardial infarction
Diabetes mellitus
Osteoporosis
Dementia
Anemia
Stroke
Cholelithiasis
Arteriosclerosis
Hypertriglyceridemia
Anosmia

Fracture
Cataract
Atrial fibrillation
Neuropathy
Seizures
Parkinsonism
Disease
Control
Years
Age
Time
Blood
Weight
Heart
Mass
Insulin
Related
Bone
Ratio
Risk of
Infarction
Pressure
Cases
Well
Months
Syndrome
Glucose
Diseases
Population
Activity
Cell
Function
Diagnosis
Follow
Pain
Artery
Serum
Failure
Blood pressure
Follow-up
Cholesterol
Primary
Vascular
Death
Events
Severe
History
Plasma
Protein
Brain
Disorders
Obese
Expression
Dose
Days

Reduction
Tissue
Depression
Drug
Within
Weeks
Presence
Diet
Heart disease
Breast
Gene
Dysfunction
Receptor
Fat
Heart failure
History of
Regression
Stress
Report
Liver
Prevention
Presence of
Coronary artery
Therapeutic
HR
Fibrillation
Many
Impairment
Exercise
Lipid
Range
Tumor
Acid
Smoking
General
Male
Cardiovascular disease
Inflammation
Assessment
Muscle
Post
Breast cancer
Percent
Often
Sex
Injury
Various
Main
Body mass index
PD
Combination
Single
Hip
Defined
Female

Disorder
Postoperative
Family
Infection
Available
Risk factor
Sensitivity
Metabolism
Likely
Administration
Result
Parameters
LDL
Volume
Provide
Vitamin
Systolic
Lipoprotein
Lung
People
Chest
Quality of life
Damage
Calcium
Diagnostic
Fasting
Kidney
Poor
Energy
Bleeding
Pre
HDL
Concentration
Moderate
Confidence
Neurological
Recently
Lead
Found in
Gender
Frequently
Coronary artery disease
Acute myocardial infarction
MRI
AD
Ischemia
Hearing
Joint
Affected
Prevent
Bypass
Body weight
Hours
Exposure
Stenosis
White

Nerve
Caused by
Physical
activity
Prognosis
Process
Angiotens
in Balance
DNA
Complication
Tolerance
Hormone
Enzyme
Understanding
Inhibition
Medication
Event
Evidence of
Animals
Proteins
Rheumatoid arthritis
Arm
Skin
Order
Toxicity
Lesion
Alcohol
M2
Onset of
Coronary heart disease
Education
Community
Platelet
Died
Affect
Absence
Approximately
Postmenopausal
SD
Currently
Carcinoma
Prostate
Bilateral
Psychological
Twenty
Pathological
Thrombosis
Mineral
Resonance
Atrophy
Unstable
Absence of
Symptom
Knee
Neck
Transient

Vitamin d
Ischaemic
Kinase
Binding
Head
Aspirin
Peptide
Ratios
Operation
Middle
Hemoglo
bin
Radiation
Plaque
Functions
Change in
Antihypertensive
Memory
ECG
Problem
Little
Center
Injection
Disability
Percutaneous
Vein
Replacement
Oxygen
Hemorrhage
Attention
Asymptomatic
Heart rate
Occlusion
Fatigue
Iron
Spine
Past
Every
Materials
Line
Plus
Necrosis
Global
Sodium
Discharge
Pharmacological
Alzheimer's disease
Trauma
Triglyceride
In addition to
Marked
Proliferation
Homeostasis
Metastatic
Lung cancer
Need for

Hand
Childhood
Nervous system
Stimulation
Maternal Creatinine
Involving Medicine
Prior to
Illness
PH
Thirty
Ultrasound
Hypertrophy
Modified
Estrogen
Review of
mRNA
Biological
Marrow
Cancers
Sinus
Plays
Fold
Family history
Infarct
Physiological
Paper
Total cholesterol
Repair
Delivery
Distress
Resection
Graft
Rehabilitation
Fibrosis
Renal failure
Referred
Phenotype
Finding
Material
Nutrition
Water
Composition
Thyroid
At risk
Birth
Larger
Tool
Blood flow
Concomitant
Systolic blood pressure
Probably
Insufficiency
Upon
Angioplasty
Growth factor
Blind

X-ray
Determined by
Metastases
Susceptibility
Forty Albumin
Attack
Goal
Urine
Idiopathic
Minutes
Hip fracture
Final
Antibody
Collagen
Variation
Markedly
Rest
Headache
Premature
Repeated
Vascular disease
Fever
Secondary to
Light
Sclerosis
Ocular
Degeneration
Physician
Injuries
Intra
Initiation
Initially
Suffering
Preventive
Exposure to
Limb
CD
Colon
Chain
Understood
Possibly
Lymph
Compression
Renal disease
Tumour
Amount
Fifty
Embolism
Renal function
Visual acuity
Includes
Histological
Catheter
Twice
Poorly
Exposed

Matrix
Gene
expression
Red
Observatio
n Renin
Pulse
Shock
Velocity
Joints
Race
Interleukin
Evidence for
Falls
Block
Aorta
Great
Extent of
Healing
Mood
Aggressive
Black
Gamma
Tau
Defect
Back
Amyloid
Awareness
Excretion
Endoscopic
Diets
Wound
Rupture
One hundred
Central nervous system
Type II
Rarely
Bladder
Heparin
Smooth muscle
Cartilage
Diastolic blood pressure
Fatty acid
Confirm
Se
Skeletal muscle
Sixty
Remission
Steroid
Bowel
Dogs
Asthma
Mortality rate
Attenuated
Frontal
Motion

Address
Testosterone
Terminal Fall Oxidation Hypotension Hypoxia Obstruction Aggregation
Normotensive
Suppression
Reporting
Salt
Periods
Feeding
Deficit
Glaucoma
Aneurysm
Myocardium
Carbohydrate
Add
Er
Radical
Antigen
Appearance
Pneumonia
Accelerated
Ear
Balloon
Gallbladder
Referred to
Dr
Yr
Hepatitis
Root
Rich
Ventricle
Cause of death
Infected
Calcification
Lymphoma
Urinary tract
Potassium
Anesthesia
Essential hypertension
Molecule
As a result of
Movement
Strain
Instability
Bile
Menopause
Lens
Smaller
Hair
Concept
Face
Turnover

Extremely
Learning
Income
Leg
Organization
Employed
Filtration
Image
Platelets
Culture
Base
Seventy
Gas
Treated by
Femur
Gait
Habits
Severely
Atp
Exposed to
Autopsy
Act
Aldosterone
Chromosome
Confirmed by
Near
Alcohol consumption
Migration
Centre
Cut
Oil
Axis
Differential diagnosis
Physical examination
Changed
Fiber
Pancreas
Ventricular hypertrophy
Institution
Definition
Cardiac output
Spinal cord
Deposition
Perception
Unchanged
Resulting from
Deviation
Dopamine
Walking
Eighty
Intraocular
Angle
Mobility
Sub
Capillary

Language
Hb Orally
EEG
Consequence of
Relief
Glomerular filtration
Arterial pressure
Prophylaxis
Air
Task
Type i
Chemical
Morphology
Nurses
Alcoholic
Treated for
Equivalent
Irradiation
Ulcer
Abuse
Drinking
Schizophrenia
Depressed
Sub-
Pt
Foods
Closure
Cortisol
Disruption
Magnesium
Serum creatinine
Electron
Start
Transfer
Hippocampus
Increased by
Sign
Fusion
Enhancement
Serum cholesterol
Tuberculosis
Acceptable
Leukemia
Lymphocytes
Malnutrition
Difficulty
Retention
Unrelated
Pilot
Phosphatase
Health status
Stomach
Adulthood
Shift
Cutaneous
Androgen

Ninety
Dilatation
Saline
RNA
Aids
T1
Reductase
Temperature
AP
Hyperplasia
No evidence of
AUC
Phosphate
Glucocorticoid
Standing
Emission
Destruction
Delirium
Sampling
Inner
Erythrocyte
Nucleus
IgG
Adenosine
Fish
Haemorrhage
Juvenile
Peripheral blood
Accident
Airway
Solution
Moderately
Cerebrospinal fluid
Thickening
Ill
Wk
Tyrosine
Preferred
Heavy
Bones
Left ventricle
Constant
Easily
Vascular resistance
Differentiated
Disc
Monitor
Dental
Zinc
Planning
Force
Glutamate
Hypothyroidism
Cytotoxic
Serotonin

Hematoma
Diuretic Section
Enlargement
Adjacent
Localization
Personality
Ventilation
Nifedipine
Anatomical
Drainage
Substances
Mitral valve
The change
Parathyroid
AMP
Forearm
Speech
Lactate
SP
Instrument
Depletion
Optic nerve
Mental state
Heat
Advantage
Glutathione
Breathing
Aortic valve
Plate
Melanoma
Decreased by
Folate
Antibiotic
Oxidase
Dexamethasone
Brainstem
Strains
Estradiol
Amino acid
Aetiology
Insomnia
Incontinence
Substance
Restoration
Ion
Twice daily
Dehydrogenase
Duct
Spleen
Gestation
Liquid
Nocturnal
Located in
Contraction
Immune system
Nurse

Cavity
IGF-I
Inappropriate
Availability of
Complement
Reflex
Pi
Prosthesis
Dwelling
Vitamin e
Mucosa
Preceding
Acetylcholine
Nutritional status
Ferritin
Capsule
Battery
Today
Outside
Acetate
Norepinephrine
Lymphocyte
Ingestion
Alkaline phosphatase
Phases
Discrimination
Adaptation
Shortening
Parathyroid hormone
Junction
Immunological
Captopril
Giant
Reflecting
Lipid peroxidation
Fit
Transformation
Tension
Ethanol
Elimination
Nutrients
Tendon
Verapamil
Advanced age
Color
Milk
Clinical diagnosis
Alzheimer disease
Sensitive to
Resistant to
Total body
Propranolol
TSH
Neoplasms
Cerebral blood flow

IgM
Enalapril
Immunoglobulin
Immunity
Dietary intake
Confusion
Intestine
Calcitonin
Melatonin
Urea
PP
Reversal
Retina
E2
Constipation
Digoxin
Nitrogen
Prostaglandin
Brown
Connective tissue
Neuron
ADL
Anaesthesia
Amyloidosis
No change
Copper
Growth hormone
Cerebellum
Mac
Antidepressant
Blue
Atenolol
Sulfate
Biosynthesis
Dystrophy
Protease
Gel
Hypothalamus
Serum albumin
ACTH
Return to
Separated
Skeleton
Author
IgA
Aspartate
Driving
T-cell
Immune response
Forming
Doctor
Vaginal
Atpase
Fibroblast
Superoxide dismutase
Private

Perforation
Chloride
Systolic hypertension
T3
Probe
Stimulus
T cell
Creatinine clearance
Ganglion
Fibre
Arteritis
Vitamin c
Examined for
Major depression
RBC
Longevity
Fluorescein
Suicide
Mental status
Newborn
Organisms
Influenza
Adverse reactions
Macular degeneration
Monoclonal antibody
Polypeptide
Substantia nigra
Vaccination
O2
Catecholamine
NSAID
Amygdala
Ubiquitin
Selenium
Carnitine
Alcoholism
Progesterone
Separation
Aortic stenosis
Bile duct
Tooth
Tablets
Urinary incontinence
T4
Electrophoresis
Brain tissue
Outer
Free radical
Neovascularization
Dopa
Hydrochlorothiazide
Il-2
Cm2
Senescence
App
Epinephrine

Job
Noradrenalin
e Epithelial
cells
Neurotransmitter
Reading
Peroxidase
Cholecystitis
Phosphorus
Fluoride
Teeth
Ne
Catalase
Vaccine
Angiopathy
Fluorescent
Myelin
Globulin
Vasopressin
Atrophic
Orientation
Endurance
Hydroxylase
Age-related macular degeneration
Lithium
Attitude
kDa
Phospholipid
Bovine
HPLC
Plasma membrane
Phospholipids
Ventral
Diurnal
Pigment
Chromatin
Cytoplasm
Intelligence
LH
Disposition
Iodine
Equilibrium
Choline
Somatostatin
Prolactin
Married
Dyskinesia
BPH
Handicap
Benzodiazepine
Sun
Myosin
Hybridization
Pb
Immunization
Adrenergic receptor

Thyroxine
Corticosterone
Isoproterenol Mao
Zoster
Senile dementia
MHC
FSH
Ache
Granules
Anticholinergic
Competence
Felodipine
Stain
Retarded
Testis
Fibronectin
Elastin
Institutionalized
Acetylcholinesterase
Aluminum
Thymus
Forebrain
Thymidine
Caries
Decay
Silver
Living alone
TRH
SAM
Soleus
Down's syndrome
Theophylline
Crystallin
Hearing aid
Cyclic amp
Ketanserin
Scopolamine
Acetyltransferase
Retinal pigment epithelium
Frail elderly
Tetanus
Involution
Drusen
Adenylate cyclase
Tardive dyskinesia
CHAT
Senile cataract
Antiserum
Twitch

**Table S4. List of 105 manually curated terms describing human aging.**

Graying of hair
Muscle weakness
Facial wrinkles
Cerebral atrophy
Telangiectasia
Alopecia
Congestive heart failure
Hypertension
Cancer
Varicose veins
Cerebellar atrophy
Visual acuity decrease
Kyphosis
Hearing loss
Hypercholesterolemia
Weight loss
Urinary tract infection
Vertigo
Psychiatric symptom
Obesity
Arthritis
Cardiac arrhythmia
Edema
Ischaemic heart disease
Angina pectoris
Anxiety
Myocardial infarction
Diabetes mellitus
Osteoporosis
Dementia
Anemia
Stroke
Cholelithiasis
Hypertriglyceridemia
Anosmia
Fracture
Atrial fibrillation
Seizures
Parkinsonism
Cataract
Decreased serum IGF-1
Arteriosclerosis
Neuropathy
Cholesterol
Depression
LDL
HDL
Coronary artery disease
Acute myocardial infarction
Angiotensin
Hemoglobin
Heart rate
Fatigue

Alzheimer's
disease
Triglyceride
Renal failure
Headache
Renal disease
Renal function
Amyloid
Fatty acid
Asthma
Testosterone
Hypotension
Hypoxia
Glaucoma
Aneurysm
Calcification
Lymphoma
Potassium
Platelets
Ventricular hypertrophy
Cortisol
Magnesium
Serum creatinine
Leukemia
Lymphocytes
Glucocorticoid
Glutamate
Hypothyroidism
Mitral valve
Lactate
Ferritin
Acetate
Parathyroid hormone
TSH
Cerebral blood flow
Immunoglobulin
Calcitonin
Melatonin
Urea
Constipation
Growth hormone
Serum albumin
Progesterone
Urinary incontinence
Epinephrine
Noradrenaline
Cholecystitis
Prolactin
Dyskinesia
Thyroxine
Caries
Iron
Renin

**Table S5. List of aging hallmark terms and their synonyms.**

| | | | |
|---|---|---|---|
| Genomic instability | Genome instability | DNA damage | |
| Telomere attrition | Telomere | Telomeres | |
| Epigenetic alterations | Epigenetic alteration | Epigenetic | Epigenome |
| Loss of proteostasis | Proteostasis | | |
| Deregulated nutrient sensing | Nutrient sensing | | |
| Mitochondrial dysfunction | Mitochondria | | |
| Cellular senescence | | | |
| Stem cell exhaustion | Stem cell | Stem cells | |
| Altered intercellular communication | Inflammation | | |
